# Combination of histopathological and electromyographic patterns can help to evaluate functional outcome of critical ill patients with neuromuscular weakness syndromes

**DOI:** 10.1186/cc2925

**Published:** 2004-09-10

**Authors:** François Kerbaul, Muriel Brousse, Frédéric Collart, Jean-François Pellissier, Denis Planche, Carla Fernandez, François Gouin, Catherine Guidon

**Affiliations:** 1 Département d'Anesthésie-Réanimation Adulte, Groupe Hospitalier de La Timone, Marseille, France; 2 Service de Chirurgie Cardiaque, Groupe Hospitalier de La Timone, Marseille, France; 3 Service d'Anatomie-pathologique et de Neuropathologie, Groupe Hospitalier de La Timone, Marseille, France; 4 Service de Neurophysiologie Clinique. Hôpital la Conception, Marseille, France

**Keywords:** critical illness, electromyography, myopathy, muscle biopsy, polyneuropathy

## Abstract

**Introduction:**

The aim of the study was to describe patterns of neuromuscular weakness using a combination of electromyography and histology, and to evaluate functional outcome in patients following complicated cardiovascular surgery.

**Methods:**

Fifteen adults requiring long-term mechanical ventilation (>15 days) following cardiovascular surgery associated with postoperative complications were prospectively included. Electrophysiological and histological analyses (muscle and nerve) were performed when failure to wean from mechanical ventilation associated with peripheral neuromuscular weakness was noticed. Functional disability was evaluated 12 months after surgery.

**Results:**

Six patients had a predominantly axonal neuropathy, six presented with myopathy, and three patients had a combination of axonal neuropathy and myopathy. All of them presented with acute tetraparesis and failure to wean from mechanical ventilation. All of the study patients who received corticosteroids exhibited a myopathic pattern (with or without axonopathic changes) but never an axonopathic pattern only. Only two of the eight survivors at 12 months were not ambulatory. These two patients had no detectable compound muscle action potential on electrophysiological examination.

**Conclusion:**

The combination of electromyographic evaluation and neuromuscular histological abnormalities could help to identify the type and severity of neuromuscular weakness, in turn helping to evaluate the patient's potential functional prognosis.

## Introduction

Critical illness polyneuropathy (CIP) and myopathy are neuromuscular disorders that occur in critically ill patients [1,2]. Clinical features often consist of difficulty in weaning from mechanical ventilation, tetraparesis and muscle wasting of the limbs, with tendon reflexes absent or markedly decreased. Regarding the causes of these disorders, it has been hypothesized that systemic inflammatory response syndrome (SIRS) and sepsis, with their impact on the body's defence system, may be involved [3,4]. Associations with drugs such as neuromuscular blocking agents, steroids and catecholamines have also been suggested [3,5,6]. In addition, other factors such as malnutrition, underlying disease, immobility and antibiotics have been considered [7].

Patients with weakness acquired in the intensive care unit (ICU) are often sedated and mechanically ventilated, and have unreliable sensory and motor examinations, and so diagnosis can be quite difficult because of the prolonged sedation. [8] Electromyography is useful for identifying and localizing a lesion to a particular component of the motor unit. Using electromyography, Bolton [9] and Zochodne [10] and their colleagues were the first to identify one of the major neuromuscular causes of neuromuscular weakness in acute ill patients, namely CIP [9,10]. However, use of electromyography and clinical examination without obtaining neuromuscular biopsy findings may lead to some patients being diagnosed as having CIP only [2,11]. Therefore, such examinations may distract attention from other neuromuscular disorders that occur in the critically ill, thus leading to identification of phenomena of neuromuscular junction blockade or critical illness myopathy, or a combined picture involving both syndromes.

Because of this, and because of the lack of studies evaluating neuromuscular disorders in ICUs using a combination of histopathological (nerve and muscle) and electromyographic studies, we conducted the present study in which we monitored critically ill patients prospectively following complicated cardiovascular surgery. After inclusion in the study, serial clinical, neuromuscular biopsy and electromyographic analyses were systematically conducted. Using this method we were able to diagnose neuromuscular disorders; to identify the predominant neurogenic or myogenic pattern, or a combined picture involving both lesions; and to compare these findings with functional outcome.

## Methods

### Hospital

This prospective study was carried out in the ICU of a teaching hospital with an 11-bed surgical unit. Annually, 650 critically ill patients are admitted following cardiac surgery using cardiopulmonary bypass.

### Patients

From 1998 to 2002, 15 patients who had a complicated course (with one or more organ dysfunctions) following cardiovascular surgery were prospectively included after prolonged mechanical ventilation (>15 days) associated with tetraparesis and failure to wean. Peripheral electromyographic analysis and neuromuscular biopsy were performed in all of these patients to determine the cause of limb weakness and diaphragm dysfunction. The medical committee of the hospital approved the study, and informed consent was obtained from the relatives of the patients.

Excluded were those patients who were suspected of having pre-existing polyneuropathy because they had a diagnosis of chronic diabetes mellitus, alcohol abuse, HIV infection, or end-stage renal disease (associated with chronic haemodialysis), or had used neurotoxic medication. Patients with acute or chronic spinal cord lesion, myasthenia gravis, or Guillain–Barré syndrome were also excluded.

After the patients had been enrolled, clinical examination was performed daily during their stay in the ICU, and we assessed motor deficit, muscle wasting, sensory loss and tendon reflexes.

In these patients undergoing cardiac surgery, at ICU admission the Euroscore was caculated to evaluate risk for postoperative mortality [12]. This prognostic scoring system was developed in Europe for use in patients undergoing cardiac surgery. The score is calculated by simple arithmetic (additive model) using risk factors that were found to be robust in predicting postoperative mortality, such as age, sex, emergency surgery, preoperative left ventricular dysfunction and type of surgery. Organ failure score and presence of SIRS were noted, according to criteria presented by Bone and coworkers [13], at admission and during the clinical course [7]. The diagnosis of SIRS required the presence of two or more of the following criteria [13]: body temperature >38°C or <36°C; heart rate >90 beats/min; tachypnoea >20 breaths/min; and hyperventilation, as indicated by an arterial carbon dioxide tension <32 mmHg, leucocyte count >12 g/l or <4 g/l, or presence of >10% immature neutrophils.

Use and dosage of neuromuscular blocking agents and intravenous corticosteroids were registered daily.

Electrophysiological monitoring and neuromuscular biopsies were performed as soon as the neuromuscular disorder was recognized after the end of prolonged sedation associated with mechanical ventilation (>15 days after the onset of mechanical ventilation). Failure to wean from mechanical ventilation was characterized by inability to extubate or inefficient spontaneous ventilation through tracheostomy. Peripheral weakness affected both proximal and distal muscle groups and was defined as failure to move against gravity.

### Electromyography

Nerve conduction studies were performed with Nicolet Viking IV apparatus via percutaneous stimulation and surface recording. Quantitative concentric needle electromyography was performed in distal and proximal muscles of upper extremities (such as deltoid, muscle interosseus I, and muscle abductor pollicis brevis) and lower extremities (such as tibialis anterior and flexor hallucis brevis). Electroneurography included upper (median or ulnar) nerves and lower (peroneal or sural) nerves. In patients who could not exercise, repetitive stimulation at 20–30 Hz was also given. Diaphragmatic and phrenic nerve studies were not performed.

Electromyography reports were analyzed by the same doctor and categorized as axonal polyneuropathy or myopathy, or a combination thereof. Patients were diagnosed as having axonal sensorimotor polyneuropathy if electrodiagnostic studies revealed very low amplitude or absent sensory responses and low motor amplitudes with normal or mildly reduced conduction velocities. Patients were diagnosed as having myopathy in the setting of low or normal motor amplitudes, with relatively normal sensory responses. Short duration of motor unit potentials with normal or early recruitment with or without fibrillation potentials, or fibrillation potentials and either no firing or polyphasic motor unit potentials of normal duration were also considered to reflect myopathy.

### Combined muscle and nerve biopsies

Muscular biopsies were obtained in all 15 patients, from skeletal muscle specimens of the vastus lateralis or anterior tibialis muscles, under local anaesthesia (lidocaine 1% 100–150 mg) if necessary; these patients did not have thombocytopenia or coagulopathy. Muscle samples were first snap frozen in isopentane precooled in liquid nitrogen and stored at -80°C until examination. For routine histology, the samples were placed in formaldehyde fixative and paraffin embedded. For conventional transmission electron microscopy, specimens were fixed in 2.5% glutaralehyde in 0.1 mol/l phosphate-buffered saline, postfixed with 1% osmic acid and embedded in araldite. Semithin resin sections were stained using toluidine blue. Ultrathin sections were double stained with uranylacetate. Histoenzymology was performed in serial transverse cryostat sections (6 µm thick), stained using routine histochemical methods [14].

Sensitive nerve biopsies were obtained from sural, peroneal nerves or the sensory branch of the musculocutaneous nerve (in the distal third of the leg). Nerve samples were fixed in 2.5% glutaraldehyde in 0.1 mol/l phosphate-buffered saline, postfixed with 1% osmic acid and embedded in araldite. Semithin resin transverse and longitudinal sections were stained using haematein and eosin, solochrome blue and paraphenylene diamine. Ultrathin sections were double stained with uranylacetate and citrate. Histopathological reports and original slides were reviewed by two medical experts who were blinded to the electromyographic findings.

### Functional outcome

Follow-up data were available in all 15 patients. The end-points were death or time to ambulation without assistance. The maximal duration of follow up was 12 months. Findings were not analyzed statistically because of the relatively small numbers included in the various groups.

## Results

### Clinical features

A total of 25 patients, suffering in most cases from sepsis or SIRS following cardiac surgery, and who were undergoing long-term mechanical ventilation (>15 days), were enrolled. Ten patients were excluded because of previous alcoholic liver disease or end-stage renal failure (*n *= 4) or a previous history of neuromuscular disease (*n *= 6).

The patients' median age was 53 years (range 33–82 years), and the median Euroscore was 7 (range 1–20). All patients presented with at least one episode of sepsis or a systemic inflammatory response. Multiorgan dysfunction was diagnosed in 10 patients, with a median Multiple Organ Dysfunction Score of 3 (range 1–4; Table 1). The most common organ system failure was cardiovascular, and more than 86% of patients fulfilled criteria for heart failure. Postoperative renal dysfunction requiring continuous venovenous haemofiltration was diagnosed in nine patients (60%). The severity of weakness and variability in reflex abnormality were noted. Patients were noted to be weak 15–40 days after ICU admission. Because patients received neuromuscular blocking agents and sedatives, the exact time of onset of weakness was usually not possible to determine. Of the patients studied, 50% presented with asymmetric tetraparesia, predominantly involving the legs. The other 50% had global tetraparesia with much reduced muscle tone (Table 1). Five patients were areflexic, seven had hyporeflexia and three had normal tendon reflexes. All patients grimaced in response to painful stimuli, but sensory testing was initially unreliable in most. One patient was transiently encephalopathic.

Table 2 summarizes clinical diagnoses and medications affecting the neuromuscular system in these patients with neuromuscular weakness syndromes. Patients in all groups received muscle relaxants; only two were used in our unit during the study period – vecuronium and atracurium. The groups included patients who received large doses of muscle relaxants as well as patients who received none. Muscle relaxants were used in 66% of the patients included in the study; 20% of these received muscle relaxants for less than 24 hours. Six patients received intravenous corticoids; four of these patients had undergone transplants and the other two patients were asthmatic.

### Electrodiagnostic testing

The spectrum of neuromuscular causes of weakness, along with electromyographic localization and neuromuscular biopsy findings, is summarized in Table 2. The two most common causes of weakness in these patients were polyneuropathy or myopathy in isolation. Six patients presented with neuropathy, three of whom had sensory motor neuropathy characterized by reduced sensory and motor action potential amplitudes. Six other patients presented with acute myopathy, characterized by sensory and motor action potentials in the normal range or partially reduced. Three patients presented with CIP associated with myopathy. Motor unit potentials were usually reduced, with pathological fibrillation potentials associated.

### Muscle and nerve pathology

**Figure 1:**
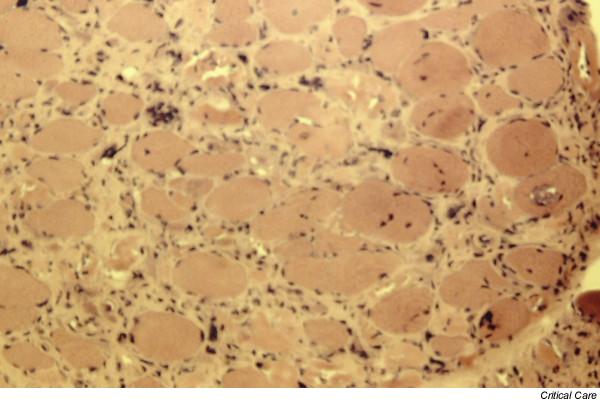
Cryostat cross-section of a biopsy specimen from patient 10 from the musculus vastus lateralis with acute necrotizing myopathy. Necrotic vacuolated and regenerating muscle fibres are present. Endomysial connective tissue is increased. Frozen section, stained with haematein and eosin, magnification 200×.

**Figure 2:**
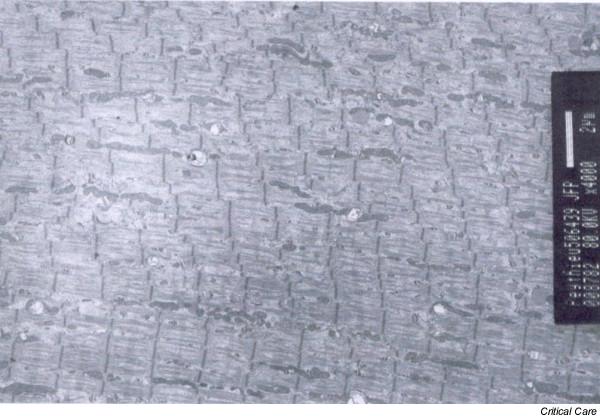
Electron microscopic changes in muscular fibres associated with a pronounced myosin heavy chain depletion. Near total loss of thick filaments is seen in the A band (barr = 2 µm).

**Figure 3:**
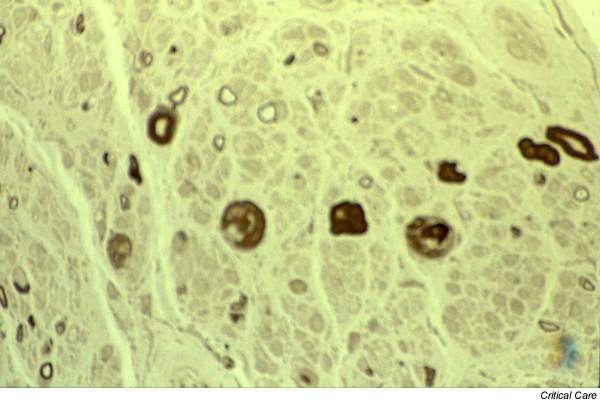
Cross-section of a nerve biopsy specimen from patient 4 exhibiting severe axonal neuropathy. There is loss of myelinated nerve fibres. Some large myelinated fibres show degenerating myelin ovoids. Secondary demyelination is seen in rare nerve fibres. Clusters of Schwann cells without nerve fibres are increased. Resin section, stained with paraphenylene diamine, magnification 115×.

A range of histopathological abnormalities was identified in the neuromuscular biopsies from the 15 patients (Table 2). The most common muscle abnormality was diffuse atrophy of fibre types I and II (14 out of 15 patients) associated with acute necrosis (12 out of 15 patients; Figs 1 and 2). There were abnormalities in the nerve biopsy from most patients (Fig. 3; except those with acute myopathy); these included axonal degeneration and demyelinating lesions. In some patients (patients 5, 7 and 8), analysis of neuromuscular biopsy revealed myopathic lesions associated with necrotic and atrophic fibres. This was associated with a huge reduction in sensory or motor action potential amplitude. Ten patients received neuromuscular blocking agents, five of whom exhibited an acute myopathy and the other five a peripheral neuropathy. However, two patients who did not receive any muscle relaxant developed a neuropathic pattern (with or without myopathic lesions). All patients who received corticosteroids exhibited a myopathic pattern (with or without axonopathic lesions) but never an exclusively axonopathic pattern.

The overall mortality rate was 40%, with a further patient dying on the ward after ICU discharge, giving a hospital mortality of 46%. Among the eight survivors at 12 months, two patients were not ambulatory (patients 2 and 8; Table 3). Compound muscle action potentials (CMAPs) were undetectable in these two patients on electrophysiological examination (Table 2).

## Discussion

Although acute neuromuscular weakness appears prevalent among patients on prolonged mechanical ventilation, few prospective studies have been reported that include both electrophysiological and histological patterns [5,15,16]. Furthermore, nerve biopsy findings are absent, even in recent prospective studies [5]. Our study is among the first to perform electromyography and obtain neuromuscular biopsies prospectively for all patients included. The neurophysiological abnormalities identified were of three types, namely CIP alone, acute myopathy and mixed neurogenic and myogenic disturbances, and they developed in a group of long-term mechanically ventilated patients who had undergone cardiovascular surgery. This type of severe and disturbing complication following cardiac surgery has been described previously [6,17], and led to an evaluation of hypothetical risk factors for such neurological disorders.

It is also widely believed that the development of critical illness neuropathy is invariably associated with multiple organ failure, sepsis and SIRS [3,6,8,9]. Thus, CIP probably represents an organ failure caused by sepsis and SIRS, presumably as a result of the same basic mechanisms that lead to multiple organ dysunction, including inflammation, thrombosis, apoptosis and oxidant injury [18]. In the present study, however, peripheral neurological changes occurred in a few patients who did not fulfill accepted objective criteria for sepsis or single organ failure. This observation was previously reported in four series of patients with respiratory failure [7,15,19,20]. As might be expected, in the present study of long-term mechanically ventilated patients following cardiovascular surgery, sepsis and multiple organ failure were common but did not seem to be a prerequisite for the development of acute neuromuscular weakness, the cause of which remains unclear [18].

It was previously suggested that use of neuromuscular relaxants is associated with neuromuscular disorders in the ICU [21-24]. Possible mechanisms include persistent effects of these drugs or their active metabolites, pharmacological denervation hastening muscle atrophy, or association of these drugs with intravenous corticosteroids or aminoglycosides [22,24]. Vecuronium and its steroid components were also implicated as a cause of weakness [25]. In our study atracurium was also administered in patients with prolonged neuromuscular weakness, although it has no steroidal component and there is no accumulation of this molecule in the event of kidney or liver failure. However, we are unable to conclude that neuromuscular relaxants predispose to the development of neuromuscular disorders in general, or any type of neuromuscular disease in particular, because of the lack of a control group. Other observational studies failed to identify neuromuscular relaxants as possible additional risk factors [20,31].

The link between use of aminoglycosides and CIP was previously reported [26,27]. In the present study 66% of patients presenting with CIP received intravenous aminoglycosides, but only five patients out of 15 received intravenous aminoglycosides. Therefore, intravenous use of aminoglycosides may be another measure of severity of sepsis and multiple organ failure.

Steroid administration appears to be associated with muscular lesions, regardless of association with neuropathy. All study patients who received high doses of corticosteroids (cumulative equivalent dose >1000 mg methylprednisolone) exhibited a myopathic pattern (with or without associated axonopathic lesion) but never an exclusive axonopathic pattern. This finding supports the deleterious effect of corticosteroids predominantly on the muscles, as suggested by several studies [5,28-32].

Electromyographic abnormalities and neuromuscular histology patterns were concordant in 13 patients out of 15. In the two discordant cases, electromyographic examination failed to show any myogenic component, whereas muscle histology suggested severe myopathy with necrotic fibres and vacuolization zones. This lack of complete agreement between neurophysiological testing and muscle histology has already been noted by Coakley and colleagues [20]. Some authors have also indicated that it could be difficult to differentiate myopathy from axonal motor neuropathy via electromyographic analysis alone, especially in unconscious patients [2,5]. Thus, in the absence of systematic muscle biopsy, some patients can be misdiagnosed with myopathy when motor axonopathy is present and, as shown in the present study, it is conceivable that some patients have both myopathy and neuropathy [4]. The combination of neurophysiological testing with neuromuscular histology could therefore help in the precise identification of the type and severity of neuromuscular weakness, and may lead to a better understanding of the causes and consequences of neuromuscular weakness [5].

There was a high mortality rate in patients with acute neuromuscular weakness. Patients who died did so as a result of their underlying diseases and not from neuromuscular affection. These findings are similar to those from previous studies that reported on neuromuscular abnormalities [4,33]. In fact, the mortality rate and functional prognosis were similar between survivors with acute myopathy and those with neuropathy. However, of the three patients with combined neurological and muscular lesions, two died and the third survived but with severe functional disability.

Survivors from critical illness have sustained impairments in physical function and health status, even after 1 year of recovery [34-36]. In our study, of the eight survivors at 12 months only two were not ambulatory (patients 2 and 8). These were the only two survivors in whom CMAPs were undetectable on electrophysiological examination. This electrophysiological finding attests to the severity of the axonopathy and may be a predictor of prolonged functional disability. However, because of the relatively small number of patients in this group, additional studies are necessary to confirm this clinical finding.

Certain limitations of present study are worthy of mention. This prospective study evaluated electrophysiological and histological neuromuscular patterns in 15 ICU patients with prolonged mechanical ventilation after a complicated course following cardiovascular surgery. However, because the lack of control group and the small numbers of patients included, we were not able to determine precisely the risk factors for each pathology. Furthermore, as in previous studies [20,37], we were unable to determine exactly the time of onset of weakness. Therefore, electrophysiological and histological analyses were not performed at the same time point for all patients.

## Conclusion

Neurophysiological and neuromuscular histological abnormalities associated with acute neuromuscular weakness were identified in mechanically ventilated patients in the ICU who had undergone cardiovascular surgery. Such patients are assumed to present with reversible neurological damage, although in a proportion this damage could be irreversible. Among survivors the absence of CMAPs on electrophysiological examination could suggest prolonged functional disability. Because of the lack of sensitivity of clinical examination in such patients, combined electromyographic diagnosis and neuromuscular abnormalities on histology could help to identify the type and severity of neuromuscular weakness, and the functional prognosis.

## Key messages

• 	In patients undergoing neuromuscular weakness syndrome following cardiovascular surgery, the combination of electromyographic evaluation and neuromuscular histological abnormalities could help identify type and severity of these neuromuscular weakness, in turn helping to evaluate more precisely the patient's functional prognosis.

## Competing interests

None declared.

## Abbreviations

CIP = critical illness polyneuropathy; CMAP = compound muscle action potential; ICU = intensive care unit; SIRS = systemic inflammatory response syndrome.

## Figures and Tables

**Table 1:** Summary of diagnoses and medications affecting the neuromuscular system in 15 patients with neuromuscular weakness syndromes

Patient	Age (years)	Sex	Euroscore	Primary disease	Surgery	Duration of MV (days)	Complications	SIRS	Sepsis	MOD score	Neurological presentation^a^	Neuromuscular blocking medication		Corticosteroids
													
												Muscle relaxants	Aminoglycosides	
1	59	M	3	Coronary artery disease	CABG	70	Pneumonia, kidney failure	Yes	Yes	2	Flaccid TP, stupor	Atracurium 1830 mg, vecuronium 72 mg	Tobramycin 240 mg, gentamycin 1120 mg	None
2	44	M	1	Obesity, asthma	CABG	52	Pneumonia	Yes	Yes	1	Flaccid TP	Atracurium 1980 mg, vecuronium 1640 mg	None	Methylprednisolone 240 mg
3	50	F	7	Active endocarditis	VR	24	RV failure, kidney failure	Yes	No	2	Flaccid TP	None	None	None
4	76	M	6	Coronary artery disease, hypertension	CABG	51	ARDS, septic shock	Yes	Yes	4	Flaccid TP	None	Amikacin 3200 mg	None
5	67	M	14	Coronary artery disease	CABG	40	ARDS, kidney failure	Yes	Yes	3	Flaccid TP	Atracurium 1380 mg	None	None
6	33	M	0	Bicuspid aorta	Ross procedure	25	Haemorrhagic shock, kidney failure, ARDS	Yes	Yes	4	Flaccid TP, distal amyotrophy	Atracurium 3000 mg	None	None
7	76	F	8	Hypertension, endocarditis	VR	163	Septic shock, kidney failure	Yes	Yes	3	Flaccid TP	None	Gentamycin 1000 mg	None
8	60	M	6	Coronary artery disease	HT	36	Cardiac arrest, pneumonia	Yes	Yes	2	Flaccid TP, diffuse amyotrophy	None	None	Methylprednisolone 2000 mg
9	60	M	20	Coronary artery disease	HT	120	Mucormycosis, pneumonia, kidney failure	Yes	Yes	4	Flaccid TP, diffuse amyotrophy, ROT	Atracurium 300 mg	Gentamycin 600 mg	Methylprednisolone 3000 mg
10	53	M	6	Coronary artery disease	HT	54	Pneumonia, kidney failure	Yes	Yes	4	Flaccid TP	Atracurium 100 mg	Tobramycin 820 mg	Methylprednisolone 8460 mg
11	57	M	9	Cardiomyopathy	HT	28	RV failure, pneumonia, kidney failure	Yes	Yes	3	Flaccid TP, ROT	None	None	Methylprednisolone 3000 mg
12	26	F	6	None	RV blast	64	Cardiac arrest, encephalopathy	Yes	No	3	Flaccid TP, ROT	Atracurium 11,000 mg	None	None
13	77	F	10	Mitral regurgitation, endocarditis	VR	45	Septic shock, kidney failure	Yes	Yes	3	Flaccid TP	Atracurium 630 mg	None	None
14	77	F	11	Hypertension, asthma	Aortic dissection	34	Pneumonia, kidney failure	Yes	Yes	2	Flaccid TP, ROT	Atracurium 7300 mg	None	Methylprednisolone 1120 mg
15	82	F	10	Hypertension	Aortic dissection	36	Pneumonia, kidney failure	Yes	Yes	2	Flaccid TP, ROT	Atracurium 3300 mg	None	None

^a^Neurological presentation after stopping sedation (approximately 72 hours). CABG, coronary artery bypass grafting; HT, heart transplantation; MOD, multiple organ dysfunction; MV, mechanical ventilation; PP, paraparesia; TP, tetraparesia; ROT, tendon reflexes abolished; RV, right ventricular; SIRS, systemic inflammatory response syndrome; VR, valve replacement.

**Table 2:** Electrodiagnostic and histopathological findings in 15 patients with neuromuscular weakness syndromes

Patient	Type^a^	Electromyography										Muscular biopsy			Nerve biopsy	
		
		Concentric needle examinatioin						Motor nerve conduction		Sensory nerve conduction		Atrophy	Necrosis	Myosin loss	Axonal degeneration	Demyelination
											
		FP	LP	Recruitment^b^												
									
				0	1	2	3	CV (m/s)	CMAP (mV)	CV (m/s)	CMAP (µV)					
1	N	+		+				Peroneal 46/ulnar 68	1/0.1			I/II			+	
2	N	+		+				Peroneal 0/median 0	0/0	Sural 0/radial 0	0/0	II	+		+	
3	N	+	+			+		Peroneal 33	0.1	Peroneal 41	10	I	+		+	
4	N	+	+					Peroneal 26/median 37	1/1	Peroneal 35/median 38	2/1	I/II			+	+
5	N	+		+		+		Peroneal 41	3	Peroneal 33	4	I/II	+		+	+
6	N	+		+			+	Peroneal 40	2	Peroneal 36/radial 48	7/48	I/II	+		+	+
7	NM	+		+				Peroneal 0	0	Peroneal 0	0	I/II	+		+	+
8	NM	+	+					Peroneal 0	0	Peroneal 0	0	I/II	+	+	+	
9	NM	+	+			+		Peroneal 38	0.1	Peroneal 35/radial 48	3/8	I/II	+	+	+	
10	M	+			+	+	+	Peroneal 36/median 34	0.8/0.8	Peroneal 52/radial 56	7/16	II	+	+	No	No
11	M			+				Peroneal 42	1.6	Peroneal 58	12	II		+	No	No
12	M	+	+					Peroneal 35	1.2	Peroneal 43/radial 43	20/22	I/II	+		No	No
13	M			+				Peroneal 39	0.8	Peroneal 42	24	II	+		No	No
14	M	+	+		+	+		Peroneal 43	1.6	Peroneal 44	12	II	+	+	No	No
15	M	+				+		Peroneal 40	1.0	Peroneal 42	7	II	+		No	No

^a^Type of neurological lesion. ^b^Recruitment (muscular contraction): 0, absent; 1, simple; 2, weak intermediate; 4, rich intermediate. Recruitment data not available for patients 4, 8 and 12. CMAP, compound muscle action potential; CV, conduction velocity; FP, fibrillation potentials; LP, slow potentials of denervation; M, myopathy; N, neuropathy; NM, neuromyopathy.

**Table 3:** Clinical outcome in 15 patients with neuromuscular weakness syndrome

Patient	Type^a^	ICU stay (days)	Clinical outcome	Ambulatory activity without assistance in <3 months	Ambulatory activity without assistance in 3–12 months	Nonambulatory	Mechanical ventilation^b^
1	N	86	TP, death (day 120)	No	No	Yes	No
2	N	80	Slight proximal TP, normal tendon reflexes	No	No	Yes	No
3	N	25	Complete recovery with normal tendon reflexes	Yes	Yes	No	No
4	N	51	TP with marked atrophies, death (day 51)	No	No	Yes	Yes
5	N	44	Complete recovery of legs, distal spasticity of arms	Yes	Yes	No	No
6	N	45	Slight proximal TP	Yes	Yes	No	No
7	NM	281	TP with marked atrophies, death (day 281)	No	No	Yes	Yes
8	NM	76	TP with marked atrophies	No	No	Yes	No
9	NM	105	TP with marked atrophies, death (day 105)	No	No	Yes	Yes
10	M	204	TP with marked atrophies, death (day 204)	No	No	Yes	Yes
11	M	57	Slight left upper MP	Yes	Yes	No	No
12	M	91	Slight proximal TP	No	Yes	No	No
13	M	45	TP with marked atrophies, death (day 45)	No	No	Yes	Yes
14	M	56	Complete recovery with normal tendon reflexes	Yes	Yes	No	No
15	M	36	TP with marked atrophies, death (day 36)	No	No	Yes	No

^a^Type of neurological lesion. ^b^Weaning from mechanical ventilation at the end of the intensive care unit (ICU) stay. M, myopathy; MP, monoparesia; N, neuropathy; NM, neuromyopathy; TP, tetraparesia.
